# Mitral chordae myxoma—chordae replacement with a premeasured gore-tex loop using a minimally invasive video-assisted approach

**DOI:** 10.1186/1749-8090-8-227

**Published:** 2013-12-11

**Authors:** Masatoshi Hata, Jan F Gummert, Jochen Börgermann, Kavous Hakim-Meibodi

**Affiliations:** 1 Department of Cardiothoracic Surgery, Heart and Diabetes Center North-Rhine Westphalia, Ruhr-University Bochum, Georgstrasse 11, 32545 Bad Oeynhausen, Germany

**Keywords:** Papillary myxoma, Mitral valve repair, Minimal invasive technique

## Abstract

Cardiac myxomas are one of the most common types of primary cardiac tumors and are associated with embolization, angina, and sudden death. Most cardiac myxomas arise from the fossa ovalis, while those that arise from the mitral valve are exceedingly rare and those that arise from the chordae are even rarer. We report the case of a 28-year-old Caucasian woman who suffered from a brain infarction. A duplex ultrasound showed no cerebrovascular stenosis or occlusion, but an echocardiogram revealed a left ventricle pedunculated mobile mass (5 mm in diameter) that was attached to the mitral valve chordae tendineae. We elected cardiac surgery to resect the cardiac tumor and to avoid further embolic events. The traditional surgical strategy—mitral valve replacement through full sternotomy—has many disadvantages, particularly for young women. Therefore we desided to use the Premeasured Gore-Tex chordal loop method followed by annuloplasty using a minimally invasive video-assisted approach. Exploration of the mitral valve showed a globular tumor involving the anterior mitral leaflet chordae tendineae, which was removed along with the involved chordae tendineae. Histopathological examination of the tissue revealed a benign polypoid myxoma. The patient had an uneventful recovery and has remained symptom-free.

Echocardiography one week after surgery showed satisfactory valve function. We believe our surgical treatment was the most appropriate option for this case and it resulted in an excellent medical outcome and improved the quality of life, including only a small lateral scar without the need for teratogenic anticoagulants.

## Background

Cardiac myxomas are one of the most common types of primary cardiac tumors and are thought to develop from primitive endothelial or subendocardial cells or from multipotential mesenchymal cells and can occur within any of the cardiac chambers. However they have a predilection to occur in the atria, particularly the left atria. Myxomas that arise from the mitral valve are exceedingly rare and those from the chordae are still rarer [1-3]. Yuan reported that of the 64 mitral myxomas described in publications (42 articles) from 2006 to June 2011, only one arose from the chordate [3].

## Case presentation

**Figure 1:**
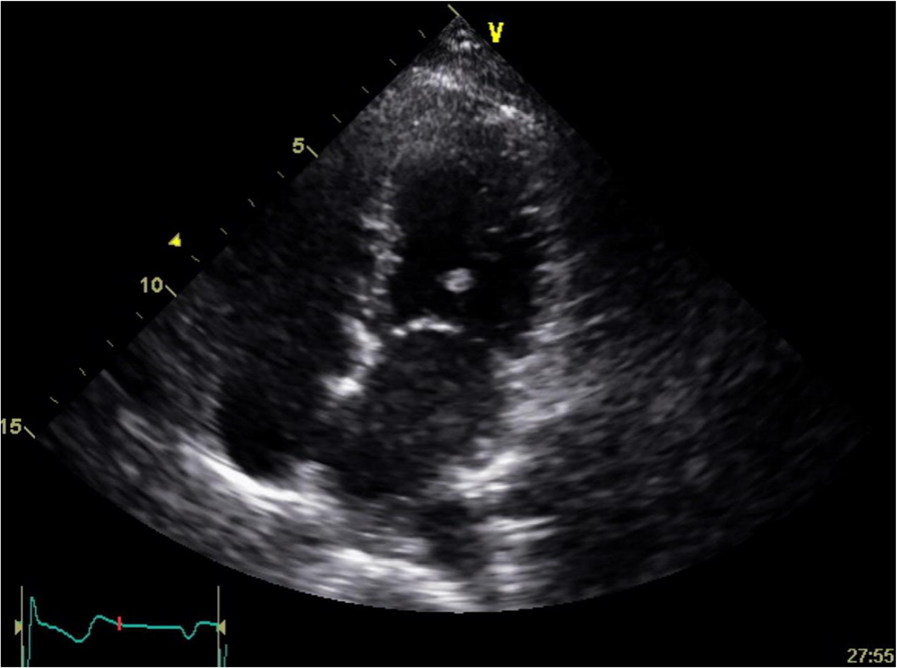
Transthoracic echocardiography showing a tumor (5 × 6 mm) on the chordae of anterior leaflet of mitral valve.

A 28-year old Caucasian woman without significant risk factors for atherosclerosis presented with headache, right hemiplegia, and right visual impairment. Magnetic resonance imaging revealed a fresh left posterior brain infarction. A duplex ultrasound showed no cerebrovascular stenosis or occlusion, but an echocardiogram revealed a left ventricle pedunculated mobile mass (5 mm in diameter) that was attached to the mitral valve chordae tendineae (Figure 1). Left and right ventricular functions were normal, and mitral valve regurgitation was mild. Because the patient did not exhibit findings indicative of having an embolic source, we elected cardiac surgery to resect the cardiac tumor and to avoid further embolic events.

### Surgical technique

**Figure 2:**
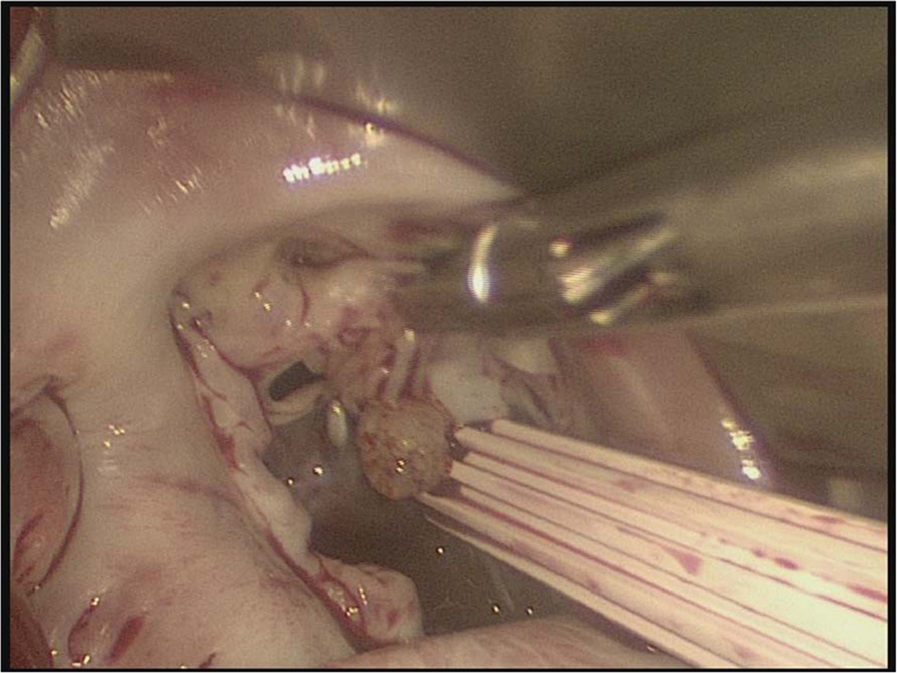
Myxoma on the chordae of anterior leaflet of mitral valve (A2, 3).

**Figure 3:**
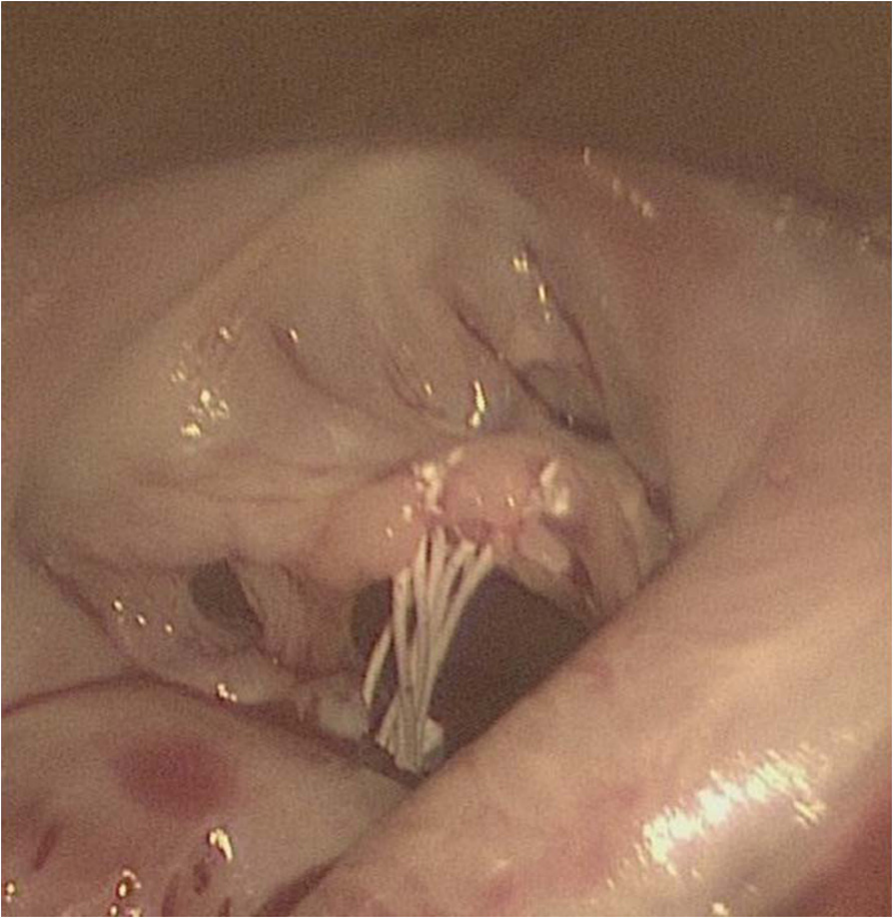
A premeasured Gore-Tex loop (18 mm) was fixed to the body of the papillary muscle and the four free loops from this suture are secured to the anterior leaflet (A2, 3) at the line of coaptation.

A CPB was instituted via the femoral arterial and venous cannulation through a 3 cm transverse incision in the right groin. The tip of the venous cannula was positioned under transesophageal echocardiographic guidance in the superior vena cava. The temperature of the patient was cooled to 34°C, and vacuum-assisted CPB was used throughout the procedure. A right lateral mini-thoracotomy (length, 5 cm) was performed via the 4th intercostal space, and the incision was placed in the submammary crease. A video camera was inserted through a 10 mm port in the right 3rd intercostal space and a transthoracic aortic cross-clamp was inserted through the 3rd intercostal space. Antegrade cold blood cardioplegia was administered directly into the aortic root and repeated every 20 min. throughout the procedure, the surgical field was flooded with carbon dioxide via the camera-port. The left atrium was opened posterior to the interatrial groove. Exploration of the mitral valve showed a globular tumor involving the anterior mitral leaflet chordae tendineae (Figure 2), which was removed along with the involved chordae tendineae. Neochordae were implanted using the loop technique. The required length of the loop was determined by measuring the distance between the correct plane of apposition on an adjacent nonprolapsing segment and the respective papillary muscle. A premeasured Gore-Tex loop (22 mm) was fixed to the body of the papillary muscle, and the four free loops from this suture were secured to the resected portion (A2,3) of the anterior leaflet at the coaptation line. The sealing-test with a saline solution showed incompetence, because of the short mitral leaflet and too small coaptation. Therefore, we removed the 22 mm Gore-Tex loop and implanted an 18 mm Gore-Tex loop with the same maneuver (Figure 3). Additionally, an annuloplasty with a 28-mm CE-Physio-ring (Edwards Lifesciences, Irvine, CA) was performed.

## Results

Histopathological examination of the tissue revealed a benign polypoid myxoma. The patient had an uneventful postoperative course and was discharged on the 7th postoperative day. An echocardiography one week after surgery showed satisfactory valve function with slight central regurgitation.

## Discussion

Cardiac myxomas are primary cardiac tumors that are thought to develop from primitive endothelial or subendocardial cells or from multipotential mesenchymal cells and can occur within any of the cardiac chambers. However they have a predilection to occur in the atria, particularly the left atria. Myxomas that arise from the mitral valve are exceedingly rare and those from the chordae are still rarer [1-3]. Yuan reported that of the 64 mitral myxomas described in publications (42 articles) from 2006 to June 2011, only one arose from the chordate [3]. Thus, our case represents an extremely rare example of such a myxoma.

Cardiac myxomas are associated with embolization, angina, and sudden death [1-3]. When they arise from the left ventricular chamber, the risk of sudden death due to embolization may increase because of the high mobility and pressure in the ventricle.

Therefore, a prompt surgical management of these tumors is necessary if no major contraindications are present. In addition, myxoma recurrence has been reported to occur at different rates in surgically-treated patients and at the site of initial localization [2]. Currently, radical resection is the gold standard for preventing myxoma recurrence. However, sometimes radical resection may handicap the patient, particularly when a heart valve is replaced.

In the young woman of our study, who could become pregnant in the future, the myxoma involved the anterior mitral leaflet chordae tendineae, necessitating complete resection of the involved chordae. The traditional surgical strategy—mitral valve replacement through full sternotomy—has many disadvantages, particularly for young women.

Anticoagulants are necessary after valve replacement with a mechanical valve, which is contraindicated in pregnancy. Thus, replacement with a biological valve is the gold standard for young women who may become pregnant in future. However, the durability of a biological valve is worth than a mechanical valve.

We have used the “Premeasured Gore-Tex chordal loop method” using a minimally invasive video-assisted approach, reported by von Oppell and Mohr [4] and is now a standard procedure used for mitral valve prolapse. Several additional repair techniques for prolapse of anterior mitral leaflet such as triangular leaflet resection, edge to edge repair and others have been developed. However, these techniques may require smaller ring size than chordae replacement for annuloplasty in cases with short mitral leaflets. Since the introduction of ePTFE as a chordal substitute, low frequency of MR recurrence after chordal replacement has been reported. Moreover, as Pfannmüller et al. have described that the premeasured loop technique is a highly reproducible method for chordal replacement and can be successfully performed using a minimally invasive approach [5]. Accordingly we use the premeasured Gore-Tex loop technique in almost all cases with mitral valve prolapse. In this case, an annuloplasty was performed because of the short mitral leaflet and small coaptation, but we consider that the annuloplasty was not necessary if the mitral valve showed an enough coaptation after chordae replacement. An echocardiography after surgery showed slight central regurgitation but it was very small and not hemodynamic relevant. New devices such as “adjustable length mitral valve chordae” are introduced and clinical trials are running at present [6,7]. Such technologies may improve the technical feasibility of chordal repair in the future.

We believe our surgical treatment was the most appropriate option for this case and it resulted in an excellent medical outcome and improved the quality of life, including only a small lateral scar without the need for teratogenic anticoagulants.

## Conclusion

We report an extremely rare case of a myxoma that arose from the mitral valve chordae tendineae. We successfully performed a minimally invasive surgery, tumor resection and replacement of only the involved parts of the chordae tendineae through a ateral mini-thoracotomy.

### Consent

Written informed consent was obtained from the patient for publication of this case report and accompanying images. A copy of the written consent is available for review by the Editor-in-Chief of this journal.

## Abbreviations

CPB: Cardiopulmonary bypass; MR: Mitral regurgitation.

## Competing interests

The authors declare that they have no competing interests.

## Authors’ contributions

MH conceived, designed and drafted the manuscript, assisted in the operation. JG was the principal operating cardiac surgeon. JB and KH were primarily involved in the clinical and scientific discussion of the case. All four authors revised the manuscript and made intellectual contributions. All authors read and approved the final manuscript.
